# A general framework for the distance–decay of similarity in ecological communities

**DOI:** 10.1111/j.1461-0248.2008.01202.x

**Published:** 2008-09

**Authors:** Hélène Morlon, George Chuyong, Richard Condit, Stephen Hubbell, David Kenfack, Duncan Thomas, Renato Valencia, Jessica L Green

**Affiliations:** 1 School of Natural Sciences, University of CaliforniaMerced, CA, USA; 2 Department of Life Sciences, University of BueaP.O. Box 63, Buea, Republic of Cameroon; 3 Smithsonian Tropical Research InstituteBox 0843-03092 Balboa, Ancon, Republic of Panama; 4 Ecology and Evolutionary Biology, University of CaliforniaLos Angeles, CA, USA; 5 Missouri Botanical GardenP.O. Box 299, St Louis MO 63166-0299, USA; 6 Department of Forest Science, Oregon State UniversityCorvallis OR 97331-2902, USA; 7 Laboratory of Plant Ecology, School of Biological SciencesAptado, 17-01-2184Pontificia Universidad Católica del EcuadorQuito, Ecuador

**Keywords:** Beta-diversity, distance–decay relationship, Poisson Cluster Process, sampling biodiversity, Sørensen index, spatial aggregation, spatial turnover, species-abundance distribution, species–area relationship, tropical forests

## Abstract

Species spatial turnover, or *β*-diversity, induces a decay of community similarity with geographic distance known as the distance–decay relationship. Although this relationship is central to biodiversity and biogeography, its theoretical underpinnings remain poorly understood. Here, we develop a general framework to describe how the distance–decay relationship is influenced by population aggregation and the landscape-scale species-abundance distribution. We utilize this general framework and data from three tropical forests to show that rare species have a weak influence on distance–decay curves, and that overall similarity and rates of decay are primarily influenced by species abundances and population aggregation respectively. We illustrate the utility of the framework by deriving an exact analytical expression of the distance–decay relationship when population aggregation is characterized by the Poisson Cluster Process. Our study provides a foundation for understanding the distance–decay relationship, and for predicting and testing patterns of beta-diversity under competing theories in ecology.

*Ecology Letters* (2008) 11: 904–917

## INTRODUCTION

One of the most widely used relationships in spatial biodiversity studies is the distance–decay, which describes how the similarity in species composition between two communities varies with the geographic distance that separates them. This relationship received the early interest of Whittaker in his seminal study of vegetation in the Siskiyou mountains (Whittaker 1960, 1972) and Preston when examining the Galapagos flora (Preston 1962). The distance–decay relationship became increasingly popular after Nekola & White (1999) formalized its ability to describe, compare and understand biodiversity patterns. Considered one of the few ‘distributions of wealth’ characterizing communities (Nekola & Brown 2007), distance–decay curves have now been studied across a wide range of organisms, geographic gradients and environments (Nekola & White 1999; Condit *et al.* 2002; Tuomisto *et al.* 2003; Green *et al.* 2004; Novotny *et al.* 2007; Qian & Ricklefs 2007; Soininen & Hillebrand 2007).

There are many reasons to explain the success of the distance–decay relationship in ecology. Data required to plot the distance–decay curve are readily obtained by sampling at local scales across a landscape, making large-scale biodiversity studies empirically tractable (Harte *et al.* 1999; Condit *et al.* 2002; Green *et al.* 2004; Krishnamani *et al.* 2004). Because the distance–decay relationship reflects patterns of spatial distribution and autocorrelation, it is likely sensitive to key spatial processes such as dispersal limitation, making it a powerful tool for testing mechanistic ecological theories (Chave & Leigh 2002; Condit *et al.* 2002). Even in the absence of theoretical derivations, distance–decay data can be used to understand the forces driving community turnover patterns such as dispersal limitation and environmental heterogeneity (Tuomisto *et al.* 2003; Ferrier *et al.* 2007; see Legendre *et al.* (2005) and Tuomisto & Ruokolainen (2006) for discussion of statistical approaches). Finally, the recent incorporation of species’ evolutionary history in distance–decay approaches offers a novel perspective for investigating the spatial turnover of phylogenetic composition across landscapes (Ferrier *et al.* 2007; Bryant *et al.* in press).

Despite a longstanding interest in the distance–decay relationship, its theoretical foundations remain poorly understood. The first theoretical derivation of the distance–decay relationship was based on dimensional analyses and the assumption of fractal species’ spatial distributions (Harte & Kinzig 1997; Harte *et al.* 1999). More recent analyses stemming from the neutral theory of biodiversity provide predictions for the distance–decay relationship in an environmentally homogeneous landscape, under the assumption that species are demographically identical (Hubbell 2001; Chave & Leigh 2002; Condit *et al.* 2002). However, a theoretical framework for the distance–decay relationship free of assumptions about the spatial organization of individuals or community dynamics is still lacking. Such a general framework is necessary to interpret distance–decay curves observed in nature, where no particular clustering or assembly processes can be assumed *a priori*.

Sampling theory provides a foundation for understanding the spatial scaling of diversity with minimal assumptions (McGill *et al.* 2007). Sampling theory has been used to derive scaling relationships for many macroecological patterns including the species–area and endemics–area relationships (He & Legendre 2002; Green & Ostling 2003), the species-abundance distribution (Green & Plotkin 2007) and species turnover (Plotkin & Muller-Landau 2002). Plotkin & Muller-Landau (2002) paved the way for integrating the distance–decay relationship into sampling theory by deriving the compositional similarity between two samples randomly drawn from a landscape, independent of their spatial location. However, the distance–decay relationship requires understanding how community similarity varies as a function of the geographic distance separating samples, and there currently exists no general sampling formula for this spatial pattern.

In this paper, we merge sampling theory and spatial statistics to develop a framework for understanding the distance–decay relationship. We begin by deriving a general formula for distance–decay as a function of the landscape-scale species-abundance distribution and intraspecific spatial autocorrelation. This general framework does not assume a particular type of population clustering or community dynamics. To illustrate the utility of this framework, we examine a specific model of clustering: the Poisson Cluster Process. This spatial-point process was chosen due to its mathematical tractability (Cressie 1993; Diggle 2003), its ability to reproduce species–area curves (Plotkin *et al.* 2000) and its potential to characterize the dispersal capacity of species (Seidler & Plotkin 2006). We compare our theoretical predictions to empirical data from three tropical forests with distance–decay curves that differ widely in their compositional similarity values, rate of decay and functional form. We conclude by discussing the implication of our results for biodiversity and biogeography studies.

## GENERAL FRAMEWORK

Our interest lies in the similarity between two sampled communities separated by a given geographic distance. We quantify community similarity using the incidence-based Sørensen index, which measures the number of species shared between two communities divided by the average number of species in each community. The analytical derivations outlined below could be readily adapted for other measures of similarity based on species presence/absence or abundance, but we focus on the Sørensen index because it is widely used in ecology (Magurran 2004), has been proposed as a means to estimate the species–area relationship (Harte & Kinzig 1997) and was adopted in the initial developments of beta-diversity sampling theory (Plotkin & Muller-Landau 2002).

### General sampling formula

Deriving a sampling formula for the distance–decay relationship requires knowledge about the abundance and aggregation of species within a landscape. Biodiversity sampling theory has traditionally assumed that population aggregation is invariant across species (He & Legendre 2002; Plotkin & Muller-Landau 2002; Green & Ostling 2003) or a linear function of population abundance (Green & Plotkin 2007). For generality, we relax this assumption by introducing *ξ*(*n*, *γ*), the joint probability that a given species in the landscape has abundance *n* and a set of clustering parameters *γ* (e.g. the parameter *k* of the negative binomial distribution, or the parameters *ρ* and *σ* of the Poisson Cluster Process).

Let *ψ*(*a*, *n*, *γ*) denote the probability that a species with landscape-scale abundance *n* and aggregation *γ* is present in a sample that covers a proportion *a* of a landscape. Let ψ*(*a*, *n*, *γ*, *d* ) denote the probability that a species with abundance *n* and aggregation *γ* is present in *a* situated at distance *d* from a focal individual. The expected Sørensen similarity *χ*(*a*, *d* )is:
(1)
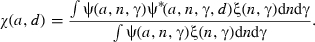

**Figure 1:**
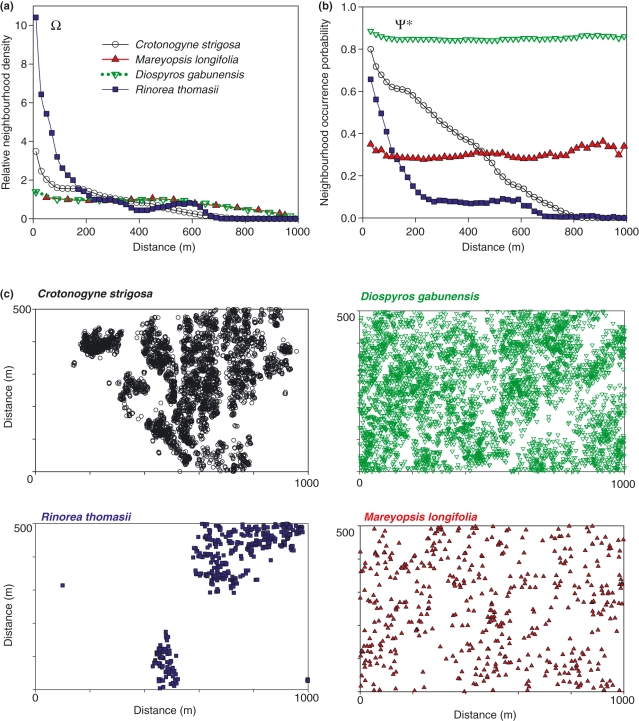
Example of (a) the relative neighbourhood density *Ω* and (b) the neighbourhood occurrence probability curves ψ* for (c) four tropical forest species in Korup National Park, Cameroon. *Ω* and ψ* are tightly linked: when a species is aggregated (i.e. *Crotonogyne strigosa*, *Rinorea thomasii*), both the relative neighbourhood density *Ω* and the neighbourhood occurrence probability ψ* are decreasing functions of distance. When a species is uniformly distributed (i.e. *Diospyros gabunensis*, *Mareyopsis longifolia*), neither *Ω* nor ψ* depend on distance. Aggregation mainly influences the shape of ψ*, and abundance its overall value. Here, ψ* is calculated in a 20 × 20 m quadrat nested in the 50-ha plot (*a = 0.0008*).

A summary of symbol notations and the theoretical underpinnings for eqn 1 can be found in Appendices SA and SB of the Supporting Information. The occurrence probability *ψ*(*a*, *n*, *γ*) is commonly used to quantify macroecological patterns such as species range size distributions and species richness in a sampling area (Gaston & Blackburn 2000). The probability ψ*(*a*, *n*, *γ*, *d* ), which we refer to as the ‘neighbourhood occurrence probability’, is novel but closely related to the classical relative neighbourhood density *Ω*(*d* ) (Fig. 1). *Ω*(*d* ) is defined as the expected density of individuals in an annulus of radius *d* and thickness *Δd* centred on a focal individual, normalized by the density of individuals in the landscape (Condit *et al.* 2000; Ostling *et al.* 2000; Wiegand & Moloney 2004). *Ω*(*d* ) is also known as the pair correlation function in spatial statistics, and is interchangeable with other correlation metrics (Appendix SB).

In Appendix SB, we derive the distance–decay relationship in terms of the correlation metric *Ω*(*d* ):
(2)
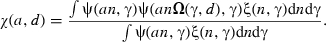

Equation 2 provides the analytical link between abundance, clustering, sample area and the decay of community similarity with distance. Although the derivation of eqns 1 and 2 require the assumption that sampling areas are relatively small compared with the geographic distance separating them (for discussion see Appendix SB), we demonstrate in *Empirical Evaluation* that these equations provide an accurate approximation over a wide range of spatial scales.

### Qualitative predictions

The general sampling formula above (eqn 2) leads to a suite of qualitative predictions that do not require assuming a specific form for the occurrence probability, spatial autocorrelation function, or landscape-scale species abundance distribution. Equation 2 does not involve the total number of species in the landscape, suggesting that the distance–decay relationship is insensitive to species richness. Equation 2 does not involve spatial correlations between species, suggesting that shuffling species in space would not affect the distance–decay relationship. Interspecific aggregation may thus only influence distance–decay curves indirectly through its influence on species’ abundances and intraspecific aggregation. Finally, the contribution of species to the integrals in eqn 2 is weighted by their landscape-scale abundance, suggesting that similarity at any distance is primarily determined by the most abundant species in a landscape and relatively insensitive to the rare ones.

**Figure 2:**
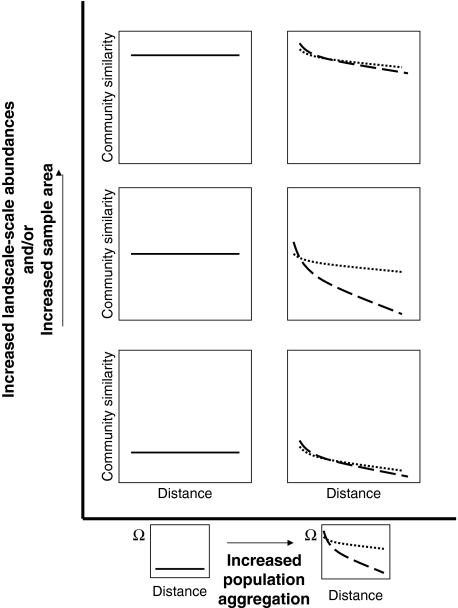
Conceptual figure illustrating the hypothetical influence of landscape-scale abundances, sampling and population aggregation on the distance–decay relationship, as suggested by eqn 2. We consider abundance *n* and sample area *a* in parallel because they are expected to have the same effect on the distance–decay relationship (community similarity at a given distance is a function of the average number of individuals in a sample *an*). *From left to right*: with comparable landscape-scale species abundances and sample area, increased aggregation (steeper decays of Ω with distance) induces steeper decays in community similarity and lower similarity values at large distances. *From bottom to top*: with comparable aggregation, increased landscape-scale abundances (or equivalently increased sample areas), induce high overall similarity. *Dashed lines:* long dashed lines reflect high aggregation, dotted lines reflect moderate aggregation. In highly aggregated communities, the distance–decay slope can be influenced by abundance and sampling at the boundaries of low and high similarity values.

Figure 2 illustrates qualitative predictions related to the influence of abundance, clustering and sample area on the distance–decay relationship. In a hypothetical landscape with even abundances and aggregation, the distance–decay formula suggests that the functional form of the relationship is primarily determined by species’ aggregation as measured by the decay of *Ω* with distance, while landscape-scale species abundances and sample area primarily influence overall similarity (Appendix SB). In biologically realistic landscapes where species differ in their abundance and aggregation, the correlation between these two variables will substantially influence the predictions above. More generally, the aggregation–abundance relationship is expected to play a major role in shaping distance–decay curves. The relative contribution of rare species to the rate of decay is expected to be more important if rare species are highly aggregated, and steep decays should occur in landscapes where the dominant species are highly aggregated.

In *Empirical Evaluation*, we test these qualitative predictions in tropical forests. We now give an example of how the framework presented above can be used to derive the distance–decay relationship when a specific type of population aggregation is assumed.

## APPLICATION: POISSON CLUSTER PROCESS

Spatial statistics have received growing interest among ecologists with the acquisition of spatially explicit data, including the establishment of large tropical forest plots around the globe (John *et al.* 2007; Wiegand *et al.* 2007). Spatial point processes provide powerful tools for characterizing aggregation. The homogeneous Poisson Cluster Process is one of the simplest, and is described in detail elsewhere (Cressie 1993; Plotkin *et al.* 2000; Diggle 2003). In short, individuals of a species are assumed to be clumped in clusters according to the following rules:

1. Cluster centres are randomly distributed in the landscape *X* according to a Poisson process with constant density *ρ.*
2. Each cluster is assigned a random number of individuals, drawn independently from a Poisson distribution with intensity μ*.*
3. The position of the individuals relative to the centre of their clusters is drawn independently from a radially symmetric Gaussian distribution *h* with variance *σ*^*2*^, namely
(3)
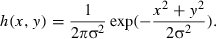

Intuitively, *ρ* reflects the density of clusters, *σ* their spatial extent and μ the number of individuals per cluster. A landscape where the homogeneous Poisson Cluster Process characterizes population aggregation consists of an independent superposition of individual species, so that interspecific spatial correlations are ignored.

The homogeneous Poisson Cluster Process provides a simple, relatively realistic characterization of population clustering (Plotkin *et al.* 2000). In nature, several processes cause clusters to form. Dispersal limitation is among the strongest, as illustrated in tropical forests by the high correlation between cluster size (as measured by *σ*) and a species’ mode of dispersal (Seidler & Plotkin 2006). The spatial distribution of clusters depend mainly on environmental heterogeneity (Plotkin *et al.* 2000; Seidler & Plotkin 2006) or secondary dispersal (Wiegand *et al.* 2007) and the parsimonious assumption that clusters are randomly distributed with constant density *ρ* may not be accurate. The degree to which the model fails in reproducing empirical patterns in nature should yield insight into the importance of incorporating heterogeneity into the Poisson Cluster model.

In Appendix SC, we derive exact analytical expressions for a species’ occurrence probability *ψ* and spatial correlation function *Ω* under the Poisson Cluster Process. From eqn 2, we deduce the distance–decay relationship in a landscape where aggregation is characterized by the homogeneous Poisson Cluster Process:
(4)
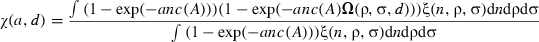

with
(5)
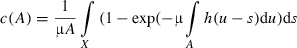

and
(6)
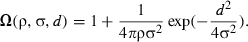

Here, *h* is given by eqn 3 (*u* and *s* represent two-dimensional coordinates in the landscape). *c* is a coefficient between 0 and 1 reflecting the deviation of the occurrence probability *ψ* from that expected under random placement. In Appendix SC, we derive the analytical link between *c* and the parameter *k* of the negative binomial distribution. Equation 4 provides the expression for the distance–decay relationship when population aggregation is characterized by the Poisson Cluster Process. The denominator in eqn 4 provides the expression for the species–area relationship.

## EMPIRICAL EVALUATION

We use data from three tropical forests to evaluate the predictions outlined above. First, we examine the qualitative predictions formulated in *General framework*, which make no *a priori* assumptions about population clustering or community dynamics. Second, we test the theoretical predictions derived in *Application: Poisson Cluster Process*. We test the accuracy of eqn 4 and the validity of the homogeneous Poisson Cluster Process as a model of clustering.

### Data

The three forest plots are part of the Center for Tropical Forest Studies network: Barro Colorado Island (Panama, 300 species), Yasuni National Park (Ecuador, 1132 species) and Korup National Park (Cameroon, 494 species). Within the 50-ha plot in Korup National Park and Barro Colorado Island, and the 25-ha plot in Yasuni, every stem > 1cm at breast height has been spatially mapped and identified to species. Detailed description of the plots and references are available on the CTFS web site http://www.ctfs.si.edu/doc/plots.

### General framework

To evaluate the general sampling formula (eqn 2) qualitative predictions, we first examine empirical distance–decay patterns in tropical forests using a sub-setting approach similar to Nekola & White (1999). We divide species into classes based on their landscape-scale abundance or degree of population aggregation (aggregation is measured using the *Ω* statistic in the 0–10 m distance class *Ω*_0–10_ following Condit *et al.* (2000)). We then compare distance–decay relationships among various subsets of the data (e.g. subsets containing mostly dominant species or highly aggregated species). Second, we compare distance–decay relationships obtained in each forest with different sampling areas, ranging from *A* = 0.0004 ha to *A* = 6.25 ha.

**Figure 3:**
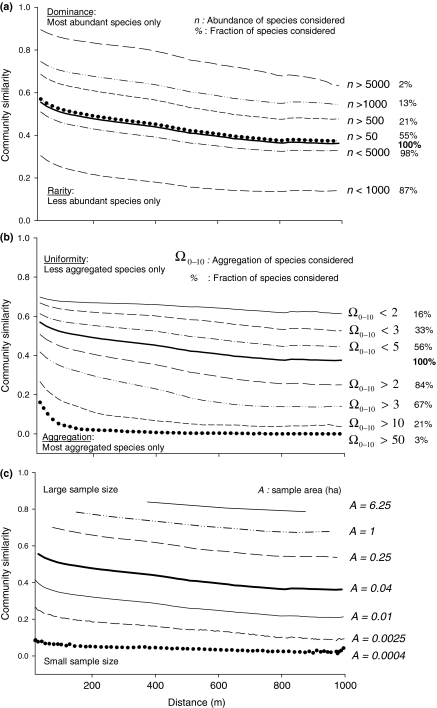
Influence of landscape-scale abundance, population aggregation and sampling on the distance–decay relationship in Korup. (a) An increasing proportion of the rarest (lines going up) or most abundant (lines going down) species are removed from the forest. Removing species with fewer than 50 individuals corresponds to considering only 55% of the landscape-scale species pool, yet this removal has very little effect on the relationship. At the other side of the spectrum, removing only 2% of the most abundant species substantially affects overall similarity. (b) An increasing proportion of the most aggregated (lines going up) or least aggregated (lines going down) species is removed from the forest. Only species with > 50 individuals are considered (Condit *et al.* 2000). (c) Sample area substantially influence rate of decays only at the smallest sample area. In (a) and (b), distance–decay plots correspond to 20 × 20 m samples nested in the 50 ha plot (*A = 0.04* ha, *a = 0.0008*). See Appendix SD for similar results in BCI and Yasuni and log-linear plots emphasizing the effect of aggregation.

Similar results, consistent with our qualitative predictions, are found in the three forests (see Fig. 3 for results in Korup, and Appendix SD for results in BCI and Yasuni). The distance–decay relationship is mainly driven by the most abundant species in a forest, and is relatively insensitive to the rare ones (Fig. 3a). The functional form of the distance–decay relationship is largely controlled by population aggregation (Fig. 3b). Finally, landscape-scale abundances and sample area influence overall similarity, rather than the rate of decay (Fig. 3c). Although these results are expected from our qualitative predictions, two caveats are in order. First, as we show below, the aggregation metric *Ω*_0-10_ is correlated with landscape-scale abundance in these tropical forests, making it difficult to infer the independent influence of aggregation versus abundance in shaping the distance–decay curves of subcommunities. Second, as illustrated in Fig. 2, sample area and landscape-scale abundances could have a stronger influence on the slope of the distance–decay relationship in landscapes where the degree of aggregation is higher than the forests studied here.

### Application: Poisson Cluster Process

Here we test the accuracy of our analytical derivations (eqn 4) using simulations, and the ability of the homogeneous Poisson Cluster Process to reproduce distance–decay relationships observed in nature. The homogeneous Poisson cluster assumptions may not precisely reflect population aggregation in the forests. BCI is a forest with relatively homogeneous environment and many generalists, where these assumptions are justified. Yasuni and Korup support several habitat types that may influence species clustering patterns in an inhomogeneous way. In *Distance–decay relationships*, we evaluate the relevance of the Poisson Cluster assumptions in the forests.

#### Clustering in tropical forests

**Figure 4:**
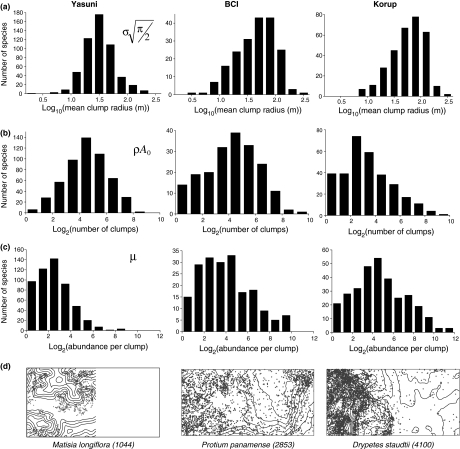
Distributions of clustering parameters estimated by the Poisson Cluster Process (a) The distributions of mean clump radius 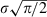 appear log-normal (in Yasuni) to right-skewed log-normal (in BCI and Korup); plotted on a linear scale, they are characterized by left-skewed shapes similar to those observed by Plotkin *et al.* (2000) (their fig. 5; see Appendix SE). (b–c) The distributions of number of clumps *ρA*_0_ and number of individuals per clump μ vary greatly between forests: species with few clusters and many individuals per cluster are common in Korup, but scarce in Yasuni, where species tend to be clumped in more clusters with fewer individuals. (d) Topographic maps and typical spatial distributions for trees in Yasuni, BCI and Korup.

**Figure 5:**
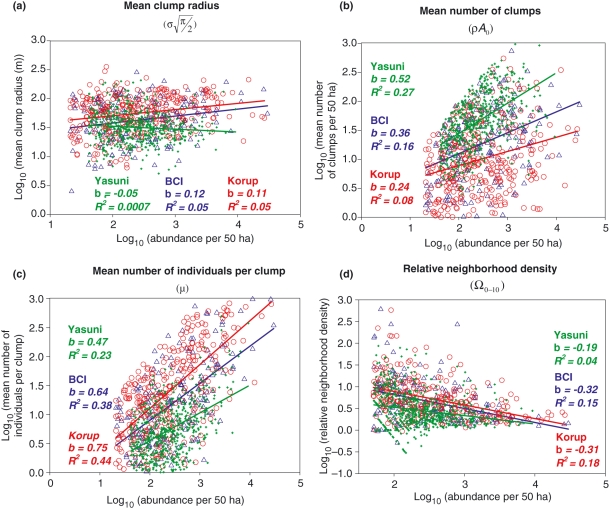
Dependence of (a) the mean clump radius 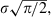
(b) the number of clumps *ρA*_0_, (c) the mean number of individuals per clump μ and (d) the relative neighbourhood density *Ω*_*0-10*_ on a species’ abundance *n*. All correlations are significant (Spearman test, *P < 0.05*); *b*-values correspond to the slope of the log–log regression of the parameters against abundance.

We fit the Poisson Cluster Process to spatial point data for each species in BCI, Korup and Yasuni (see Appendix SE for parameter estimation details). Figures 4 and 5 reveal important differences about population aggregation patterns among the three forest plots. In Yasuni, conspecifics tend to be grouped into small (Figs 4a and 5a) and numerous (Figs 4b and 5b) clusters containing few individuals (Figs 4c and 5c). This trend gets stronger as abundance increases. In Korup, conspecifics tend to be grouped into large and sparse clusters containing many individuals. These differences may be explained by differences in the ecology of each site. Korup is divided into two distinct regions: one steep/rocky ridge and one muddy/flat valley. Species tend to specialize in one of the two terrains, forming few large densely populated clumps (Fig. 4d). Environmental heterogeneity such as gullies, steep slopes, flats, wet and dry sections within these terrains likely form nested clusters. The Poisson Cluster Process, designed to characterize one scale of aggregation only, may fail to detect the smaller nested clumps. In Yasuni, valleys and ridges also constrain the spatial repartition of flora, but they are narrower and less dramatic than in Korup, the soil is more homogeneous, and the species are more generalists (Valencia *et al.* 2004). As a result, species typically have numerous small clusters spanning the entire plot.

The correlation between clustering and abundance is fundamental in shaping distance–decay curves. Understanding this correlation can also help in formulating hypotheses on the origin of rarity in tropical tree communities (Hubbell 1979). There is no consensus on how aggregation scales with abundance: positive (He *et al.* 1997), negative (Hubbell 1979; Condit *et al.* 2000) and insignificant (Plotkin *et al.* 2000) relationships have been proposed. The correlation between aggregation and abundance depends on how aggregation is quantified. Measuring aggregation in the forests using the mean clump size *σ* (Fig. 5a), we find a weak correlation between aggregation and abundance, consistent with Plotkin *et al.* (2000). Using the neighbourhood occurrence probability *Ω*_0-10_ (Fig. 5d), we find a negative correlation between aggregation and abundance, consistent with Condit *et al.* (2000). This disparity can be understood from the expression for *Ω* under the Poisson Cluster Process (eqn 6) (see Appendix SF for details). In brief, *Ω* reflects both the size of clusters (*σ*), which is independent of abundance (Fig. 5a), and their density in the landscape (*ρ*), which is correlated with abundance (Fig. 5b). Analysing the three Poisson Cluster Process parameters (*σ*, *ρ* and μ) in concert provides the most comprehensive view of the abundance–aggregation relationship. A consequence of the observed high correlation between *ρ* and *n* relevant to our distance–decay analyses is that aggregation parameters in eqn 4 cannot be assumed invariant across species, thus justifying the consideration of the joint distribution *ξ*(*n*, *γ*).

#### Distance–decay relationships

**Figure 6:**
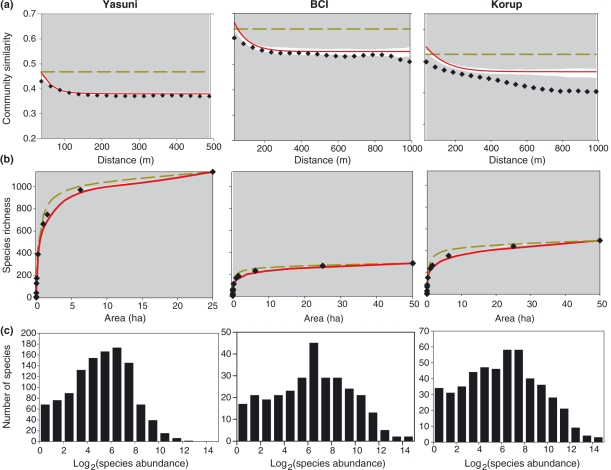
Comparison of theory with data in Yasuni, BCI and Korup (a) distance–decay curves reported for 25 × 25 m samples (*A = 0.0625* ha), (b) species–area curves, (c) species-abundance distributions. The diamonds represent observed data. The red solid lines represent curves predicted by the Poisson Cluster Process (eqn 5). The white area represents the 95% confidence intervals produced by simulation of the Poisson Cluster Process. The green dashed lines represent curves predicted when assuming random placement (Appendix SF). The sensitivity of the results to sample area is presented in Appendix SF.

Using the data parameterized above, we test eqn 4 and the ability of the homogeneous Poisson Cluster Process to reproduce distance–decay relationships. Figure 6 illustrates the results obtained by sampling 25 × 25 m quadrats from the landscape. Results for a wider range of sampling areas are presented in Appendix SF. To put our results in context with previous studies (Plotkin *et al.* 2000), we also examine species–area relationships. To test eqn 4, we compare the predicted distance–decay and species area curves to the mean and 95% confidence envelope obtained by simulations of the process (see Appendices SE and SF for details). We find that predictions and simulations agree, with only a slight overestimation for community similarity at small distances, showing that approximations made in eqns 1 and 2 are relevant, and demonstrating the accuracy of the framework and specific derivations under the process.

To test the ability of the Poisson Cluster Process to reproduce distance–decay and species–area relationships, we compare the curves directly obtained from the raw data to those predicted by eqn 4, and we use simulations to test for the significance of the results (see Appendix SF for statistical methods). Consistent with previous studies (Plotkin *et al.* 2000), we find that the Poisson Cluster Process accurately reproduces observed species–area relationships (*P > 0.05*). The accuracy of the Poisson Cluster Process to reproduce observed distance–decay relationships is less straightforward. The hypotheses that aggregation can be modelled with the process is rejected in the three forests (*P < 0.05*), except in Yasuni and BCI with 25 × 25 m sample areas. The process tends to overestimate similarity values in the forests for small sample areas, and to underestimate them for larger sample areas (see Appendix SF). In Yasuni and BCI the Poisson Cluster Process is nevertheless a reasonable first approximation of clustering patterns. In Korup, however, similarity values are largely overestimated at any distance-class and all but the 100 × 100 m sample area.

Korup appears to be an outlier: population aggregation in this forest is not well characterized by the simple homogeneous Poisson Cluster Process. The inability of Poisson Cluster Process to reproduce distance–decay relationships in Korup probably lies in its inability to reproduce species’ spatial autocorrelation (decays of *Ω* with distance). The species–area relationship, which does not reflect *Ω*, is well reproduced by the process. Species’ spatial autocorrelation in Korup may be poorly reproduced as a result of species having more than one scale of aggregation, as suggested by the ecology of the site (see *Clustering in tropical forests*). The shape of the distance–decay curve in Korup supports this hypothesis: the curve is characterized by two distinct range of distances where the decay is steeper (0 ≤ *d* ≤ 200 and 400 ≤ *d* ≤ 600), suggesting that two scales of aggregation occur in this forest.

## DISCUSSION

The distance–decay relationship reflects how diversity is spatially distributed and has consequences for conservation and our general understanding of community assembly. Interpreting this relationship and using it to test theories in ecology requires understanding how patterns in the distribution and abundance of species influence its shape. Our general distance–decay framework provides a theoretical foundation for addressing this need. The derivation under the Poisson Cluster Process illustrates a specific application of this general framework, and the efficiency of the distance–decay relationship in falsifying theories.

### General framework

Our distance–decay framework provides a theoretical foundation for interpreting earlier analyses of beta-diversity based on empirical and simulated data. Equation 1 shows that the distance–decay curve follows from a weighted combination of species-level neighbourhood occurrence curves. This prediction is in agreement with neutral theory predictions of a ‘compound’ curvilinear distance–decay curve (Hubbell 2001). Hubbell (2001) also proposed that the initial steep decay of similarity at short distances is induced by rare species, while the following shallow decay is induced by more abundant ones. In contrast, our results (eqn 3 and Fig. 3a) support the hypothesis that rare species have a weak influence on the distance–decay relationship. These results might be specific to the incidence-based Sørensen index of similarity we considered in our study. However, Nekola & White (1999) measured community similarity with the Jaccard index and also found that removing the rare species in a landscape (measured as species with low occurrence) does not affect the slope of the relationship. We expect abundance-based metrics to be even less sensitive to the rare species since they give more weight to dominant species. The distance–decay relationship should thus be robust to the potential bias caused by sampling the most abundant species in a landscape, as is common, for example, in microbial ecology.

A central hypothesis stemming from our analyses is that the slope of the distance–decay relationship alone is a poor indicator of species spatial turnover (or *β-*diversity) and total species richness in a landscape (or γ-diversity). Understanding how turnover in community composition across a landscape relates to the rate of species gain with sampling area has been the focus of many studies (Harte *et al.* 1999; Lennon *et al.* 2001). It is commonly believed that a shallow distance–decay slope reflects a low rate of species turnover, leading to low diversity at large spatial scales. This idea was formalized by Harte *et al.* (1999) in the context of self-similarity and proposed as a means to estimate diversity at large spatial scale from the sampling of small plots (Harte *et al.* 1999; Green *et al.* 2004; Krishnamani *et al.* 2004). Our results suggest that the slope of the distance–decay relationship is a poor indicator of landscape-scale species richness, complementing previous results showing that a significant taxa–area relationship can hold even when the distance–decay relationship is flat (Woodcock *et al.* 2006), or that richness estimators based on the rate of decay in similarity perform poorly (Jobe 2008). For example, Fig. 3a shows that the slope of the distance–decay curve can be conserved even when only a small fraction of the species is considered. Figure 3b shows that the slope of the distance–decay at small spatial scales is the steepest for highly aggregated communities, also known to display the shallowest species–area slopes at this scale (He & Legendre 2002). Finally, Fig. 6 shows that the most species rich forest in our study (Yasuni), has the shallowest distance–decay slope. We suggest that steep decays characterize communities where abundant species are highly aggregated rather than communities with high spatial turnover, and that *β*-diversity is better described by overall similarity than by rates of decay. We support the idea that the focus on the slope of the relationship (e.g. Qian *et al.* 2005; Qian & Ricklefs 2007) must be expanded to include a focus on intercepts and half-distances (Soininen & Hillebrand 2007), or average similarity (Plotkin & Muller-Landau 2002).

Our analyses illustrate the superiority of the distance–decay to the species–area relationship in testing spatial ecology theories, and provide the analytical basis for deriving expectations for this relationship under competing ecological hypotheses. While species–area relationships can be derived without precise information on species-level spatial autocorrelation, we show that this information is crucial in shaping distance–decay curves (eqn 2), suggesting that distance approaches are particularly informative of spatial structure in ecological communities. Analytical derivations for species-level spatial autocorrelations exist under theories such as neutrality (in absence of speciation) (Houchmandzadeh & Vallade 2003), self-similarity (Ostling *et al.* 2000), multiscale or inhomogeneous point processes (Diggle *et al.* 2007; Wiegand *et al.* 2007). These expectations could be combined with our framework to predict the community level distance–decay relationship expected under different scenarios of spatial organization.

### Poisson Cluster Process

Specific derivations under the Poisson Cluster may inform future research aimed at understanding the role of dispersal mechanisms in shaping the decay of similarity in ecological communities. Nekola & White (1999) first noted that the mode of dispersion influences distance–decay slopes, with more vagile communities displaying a shallower decay. Hubbell's (2001) neutral theory predicts that dispersal limitation and speciation alone can drive species turnover in a homogeneous landscape. Finally, source-sink meta-communities predict a decrease in beta-diversity with increasing dispersal (Mouquet & Loreau 2003). The Poisson Cluster Process is phenomenological, not mechanistic, and should not be used as a model of community assembly (but see Potts *et al.* 2004; John *et al.* 2007). However, the parameter *σ* reflecting the size of clusters is strikingly correlated with the dispersal capacity of species (Seidler & Plotkin 2006), and is incorporated explicitly in our expression for the distance–decay relationship (eqn 4). The equation along with findings by Nekola & White (1999) and neutral theory (Hubbell 2001; Chave & Leigh 2002) suggests that strong dispersal limitation (small *σ* values) induces a steep decay in community similarity.

Combining distance–decay analyses to the Poisson Cluster Process reveals limitations of this process as a model of clustering that had not been previously demonstrated. After it was shown that tropical tree populations are spatially aggregated (Hubbell 1979; He *et al.* 1997; Condit *et al.* 2000), Plotkin *et al.* (2000) proposed that randomly distributed population clusters (i.e. the Poisson Cluster Process) could be a good model of spatial organization and showed that this model accurately reproduced species area curves in 50 ha tropical forest plots, a result reproduced in our study (Fig. 6). The comparison of the distance–decay relationships observed in the forests to those produced by the Poisson Cluster Process suggests that this process does not universally reproduce clustering patterns. Several assumptions underlying the homogeneous Poisson Cluster Process are violated in natural systems. First, the Poisson Cluster Process assumes one scale of aggregation only, while ecological processes act at multiple spatial scales (e.g. adaptation to a heterogeneous landscape, dispersal limitation, intra- and inter- specific competition, facilitation and localized pest pressure) to induce nested clustering (Levin 1992; Plotkin *et al.* 2002; Cornell *et al.* 2007; Scanlon *et al.* 2007; Wiegand *et al.* 2007). Second, the process assumes a constant density of conspecifics across the landscape, whereas abundances are known to vary widely across a species’ range (Brown *et al.* 1995). It is therefore not surprising that the Poisson Cluster Process performs better in a more homogeneous environment (e.g. BCI), or when clumps span the landscape despite environmental heterogeneity (e.g. Yasuni), than when the density of trees is inhomogeneously distributed in the landscape (e.g. Korup).

The limits of the Poisson Cluster Process outlined above should not overshadow its utility, and the benefits gained from merging this model with sampling theory. Although the Poisson Cluster Process is not mechanistic and does not always reproduce patterns accurately, considering this process allowed us to develop theoretical basis for introducing spatial statistics into *β*-diversity studies. This approach could be extended to integrate processes across spatial scales, which remains a major challenge in ecology. To capture biodiversity patterns at both small and large scales, the assumption of a constant density of individuals over space, as specified by the homogeneous Poisson Cluster Process, could be relaxed. One could consider an inhomogeneous Poisson Cluster Process (Diggle *et al.* 2007), allowing the intensity of the process to vary with environmental variables, or to follow a ‘peak and tail’ distribution (McGill & Collins 2003) with population abundance ‘hotspots’ across the landscape. Considering the Poisson Cluster Process allowed for the analytical derivation, in a common framework, of two of the most widely studied spatial biodiversity patterns in ecology. This first step towards theoretically linking the increase of richness with area and the decay of community similarity with distance offers the promise of estimating diversity at large spatial scale with feasible sampling effort.

## CONCLUSION

Our study illustrates the power of the distance–decay relationship in falsifying models, and renders the relationship analytically tractable, offering a promising framework for testing theories in ecology. Theoretical ecology has placed great emphasis on the species-abundance distribution and species–area relationship, leaving the distance–decay relationship largely ignored. Our analyses provide a unified framework for systematic analysis of spatial biodiversity patterns in relation to abundance and aggregation that may inform future research aimed at understanding how biodiversity is distributed and maintained.

## SUPPORTING INFORMATION

Appendix SA Symbol notations used in the distance–decay theoretical framework.

Appendix SB Derivations of the general sampling formula for the distance–decay relationship and related predictions.

Appendix SC Poisson Cluster Process derivations.

Appendix SD Figures relevant to empirical evaluation of *Qualitative predictions* in BCI, Yasuni and Korup.

Appendix SE Estimation and distribution of Poisson Cluster Process parameters.

Appendix SF Supplementary information relevant to *Empirical evaluation*.

Appendix SG Supplementary material references.
