# Supplementary material for: A general framework for the distance–decay of similarity in ecological communities
Source: Ecol Lett. 2008 Sep;11(9):904–17. doi: 10.1111/j.1461-0248.2008.01202.x (PMC2613237; doi:10.1111/j.1461-0248.2008.01202.x)
Supplement: Appendix SA — Symbol notations used in the distance–decay theoretical framework. [file ele0011-0904-sd1.doc]

**APPENDIX A. Symbol notations used in the distance-decay theoretical framework**

| **Symbol** | **Definition** |
| --- | --- |
| $A_{0}$ | area of the landscape |
| $S_{0}$ | total number of species in the landscape |
| *A* | area of a sample |
| *a* | sample fraction area =$\frac{A}{A_{0}}$ |
| *n* | species landscape scale abundance |
| *γ* | set of parameters reflecting a species aggregation |
| *ξ* | joint distribution of species abundance and aggregation |
| *χ* | Sørensen index of similarity : fraction of species shared between two communities |
| *ψ* | occurrence probability: probablity that a species is present in a sample |
| *ψ** | neighborhood occurrence probablity: probability that a species is present in a sample in the neighborhood of a conspecific |
| Ω | relative neighborhood density : density of individuals in an annulus around a conspecific normalized by the density of individuals in the landscape |
| *ρ* | density of clusters in the landscape under a Poisson Cluster Process |
| $\sigma$ | $\sigma\sqrt{\frac{\pi}{2}}$: mean clump radius  $\sigma^{2}$:variance of the Gaussian distribution under a Poisson Cluster Process |
| *μ* | average number of individuals per cluster under a Poisson Cluster Process |

**APPENDIX B. Derivations of the general sampling formula for the distance-decay relationship and related predictions**

**B1. Generalization of Plotin & Muller Landau’s (2002) sampling formula of beta-diversity (derivation of Equation 1)**

Consider two samples whose areas constitute proportions *a* and *b*, respectively, of a landscape. With the notations introduced in the manuscript, Plotkin & Muller-Landau’s formula for the expected fraction of species shared between two samples, also known as the classic incidence-based Sørensen index, takes the form (their Equation 10):

$\chi(a,b)=\frac{\int\psi(a,n,\gamma)\psi(b,n,\gamma)\xi(n,\gamma)dnd\gamma}{\frac{1}{2}\left[\int\psi(a,n,\gamma)\xi(n,\gamma)dnd\gamma+\int\psi(b,n,\gamma)\xi(n,\gamma)dnd\gamma\right]}$ [B.1]

$\xi(n,\gamma)=\varphi(n)\theta(\gamma|n)$, where $\varphi(n)$is the landscape scale species abundance distribution, and $\theta(\gamma|n)$is the distribution of species clustering in the abundance class *n*. Alternatively, $\xi(n,\gamma)=\theta(\gamma)\varphi(n|\gamma)$where $\theta(\gamma)$ is the distribution of species clustering and $\varphi(n|\gamma)$is the distribution of abundances in the aggregation class γ. The integrals in the numerator and denominator of Equation B.1 are integrals with respect to both *n* and *γ*. The integral other *n* goes from 0 to $\infty$. The boundaries with respect to γ depend on the measure chosen for aggregation. This formula assumes that samples are drawn from one landscape characterized by a single species-abundance distribution, but one could relax this hypotheses and consider the joint distribution of abundances in multiple biomes or habitat types (Plotkin & Muller-Landau 2002).

Equation B.1 provides the expected community similarity between any two samples randomly drawn from the landscape, regardless of their spatial location. To derive an expectation of the decay in similarity with geographic distance *d*, we introduce the “neighborhood occurrence probability”,$\psi*(a,n,\gamma,d)$, defined as the probability, for a species with landscape-scale abundance *n* and aggregation parameter *γ*, that at least one conspecific occurs in a sample area constituting a fraction *a* of the landscape situated at a distance *d* from a focal individual. When the sample size *a* is small compared to the landscape, the neighborhood occurrence probability is equivalent to the probability that at least one conspecific occurs in sample area *a* situated at a distance *d* from a non-overlapping sample of area *a* containing the species.

The expected Sørensen similarity between two non-overlapping samples whose areas constitute proportion *a* of a landscape and separated by a distance *d* is thus given by:

$\chi(a,d)=\frac{\int\psi(a,n,\gamma)\psi*(a,n,\gamma,d)\xi(n,\gamma)dnd\gamma}{\int\psi(a,n,\gamma)\xi(n,\gamma)dnd\gamma}$ [B.2]

Equation B.2 will likely break down for large sample sizes, when the occurrence probability at a distance *d* from a sample cannot be approximated by the neighborhood occurrence probability *ψ**. Equation B.2 shows that the shape of the decay curve is principally the result of the compound shapes of the curves describing the decay of neighborhood occurrence probability with distance for each species, weighted by the species occurrence probability. Interspecific heterogeneity and multiple spatial scales of aggregation will likely cause the functional form of *ψ** to vary widely between species and across geographic scales, making the distance decay relationship a compound of various curves with potentially different functional forms.

**B2. Relationship between the neighborhood occurrence probability ψ* and Ω (derivation of Equation 2)**

Here, we derive the relationship between a species’ neighborhood occurrence probability $\psi*(a,n,\gamma,d)$and a species’ occurrence probability $\psi(a,n,\gamma)$, and the spatial autocorrelation function *Ω(d)*. Recall that $\psi*(a,n,\gamma,d)$ is the expected probability that a species with abundance *n* and aggregation parameters *γ* occurs in a sample *a* situated at a distance *d* from an arbitrary individual. Consider an annulus with inner radius *d* and area $A(d)$at a distance *d* around an individual, and a sampled region in the annulus whose area constitutes proportion *a* of the landscape$A_{0}$. The fraction of annulus constituted by this sample is: $a\frac{A_{0}}{A(d)}$ , and, by definition of *Ω(d)*, he number of individuals in the annulus is: $n\Omega(d)\frac{A(d)}{A_{0}}$ . We make the reasonable assumption that clustering in the annulus can be modeled by the set of parameters *γ* that reflects clustering in the landscape. For a species with abundance *n* and set of aggregation parameters *γ*, the expected neighborhood occurrence probability in sample *a* at a distance *d* from an individual is given by:

$\psi*(a,n,\gamma,d)=\psi(a\frac{A_{0}}{A(d)},n\Omega(d)\frac{A(d)}{A_{0}},\gamma)$ [B.3]

If the occurrence probability is a function of the average number of individuals in a sample *an* (example: Poisson and negative binomial distribution, Poisson Cluster Process; counter-example: self-similarity), we can write:

$$\psi(a,n,\gamma)=\psi(an,\gamma)$$

and:

$\psi*(a,n,\gamma,d)=\psi(an\Omega(\gamma,d),\gamma)$ [B.4]

Equation B.4 is intuitive: it shows that the probability that a species with abundance *n* and clustering *γ* is present in a sample area *a* at a distance *d* from a conspecific is equivalent to the probability that a species with abundance *nΩ*(*d)* and same clustering γ is present in a sample area *a*. It follows that:

$\chi(a,d)=\frac{\int\psi(an,\gamma)\psi(an\Omega(\gamma,d),\gamma)\xi(n,\gamma)dnd\gamma}{\int\psi(an,\gamma)\xi(n,\gamma)dnd\gamma}$ [B.5]

**B3. General framework assumptions**

Equation B.2 (Equation 1 in the manuscript) makes the assumption that the probability of occurrence in a sample situated at a distance *d* from an *occupied sample* is equivalent to the probability of occurrence in a sample situated at a distance *d* from a *conspecific* (*ψ**). This assumption requires that sample sizes are relatively small.

Equation B.4 (and thus Equation B.5 and Equation 2 in the manuscript) makes the assumption that the density of individuals in a sample area *a* situated at a distance *d* of an individual is constant in the sample. Therefore, Equation 4 assumes that *a* is sufficiently small relative to *d*.

Hence, our derivations are expected to be valid for relatively small sample sizes and relatively large distances. In practice, distance-decay curves are commonly designed to study the spatial organization of community composition at large spatial scales, with small sample areas and wide geographic coverage. The accuracy of the equations is tested in Figure 6 in the manuscript and Appendix F below.

**B4. Other measures of conspecific spatial autocorrelation and their relationship to the neighborhood occurrence probability Ω**

Our derivations use the statistic *Ω*(*d)* to describe spatial autocorrelation. Here, we provide the exact analytical link between *Ω*(*d*) and two other measures of spatial autocorrelation: *1*) the correlation function *ρ*(*d*), defined as the probability that two individuals from a species are at a distance *d* from each other *2*) the Ripley’s statistic *K(d)*, defined as the number of individuals in a circle of radius *d*, normalized by the density of individuals.

Relationship between *Ω*(*d*) and *ρ*(*d*)

Consider a species with total abundance *n* in a landscape of area *A_0_*. Consider annuli with inner radius *d* and area *A*(*d*) around each individual, and let *<n*(*d*)*>* be the average number of individuals in those annuli. By definition:

$\Omega(d)=\frac{\frac{<n(d)>}{A(d)}}{\frac{n}{A_{0}}}$ [B.6]

*< n*(*d*) *>* is given by: $<n(d)>=\frac{1}{n}\sum_{i=1}^{n} \sum_{j=1}^{n} \delta(d(i,j)\in[d-\Delta d,d+\Delta d])$

Here *δ* is Dirac’s delta function ($\delta(B)=1$if statement *B* is true, 0 otherwise)

By definition, $\rho(d)=\frac{p(d)}{p}$ where *p*(*d*) is the number of pairs separated by a distance comprised between $d-\Delta d$and $d+\Delta d$and *p* is the total number of pairs.

The total number of pairs *p* is given by $p=\frac{n(n-1)}{2}$

*p(d*) is given by: $p(d)=\frac{1}{2}\sum_{i=1}^{n} \sum_{j=1}^{n} \delta(d(i,j)\in[d-\Delta d,d+\Delta d]$

Hence, $\rho(d)=\frac{1}{n(n-1)}\sum_{i=1}^{n} \sum_{j=1}^{n} \delta(d(i,j)\in[d-\Delta d,d+\Delta d]$

And $<n(d)>=(n-1)\rho(d)$

Finally:

$\Omega(d)=\frac{\frac{(n-1)\rho(d)}{A(d)}}{\frac{n}{A}_{0}}$ [B.7]

Relationship between *Ω*(*d*) and *K*(*d*)

Let *< N*(*d*)*>* be the average number of individuals in the circles of radius *d* surrounding each individual in the landscape, and let *λ* be the density of individuals in the landscape. By definition: $K(d)=\frac{1}{\lambda}<N(d)>$

In the annulus delimitated by radii *d_1_* and *d_2_*, by definition:

$$\Omega(d_{1,2})=\frac{<N(d_{2})>-<N(d_{1})>}{\lambda(\pi d_{2}^{2}-\pi d_{1}^{2})}$$

Thus: $\Omega(d_{1,2})=\frac{K(d_{2})-K(d_{1})}{\pi(d_{2}^{2}-d_{1}^{2})}$ [B.8]

and

$$\Omega(d)=\lim_{d_{1}\to d_{2}=d}\Omega(d_{1,2})=\lim_{d_{1}\to d_{2}=d}\frac{K(d_{2})-K(d_{1})}{\pi(d_{2}^{2}-d_{1}^{2})}$$

**B5. General framework predictions**

To interpret Equation B.5 (Equation 2 in the manuscript), it may be more intuitive to consider its discrete equivalent:

$\chi(a,d)=\frac{\sum_{i=1}^{S_{0}} \psi(an_{i},\gamma_{i})\psi(an_{i}\Omega_{i}(d),\gamma_{i},d)}{\sum_{i=1}^{S_{0}} \psi(an_{i},\gamma_{i})}$ [B.9]

Here, $S_{0}$ is the total number of species in the landscape, and *i* stands for species *i*.

In a hypothetical community where all the species have same abundance *n* and aggregation γ,$\chi(a,d)=\psi(an\Omega(\gamma,d),\gamma)$, so that as a first approximation, the dependence of similarity on abundance, aggregation, and sampling should follow that of the neighborhood occurrence probability $\psi(an\Omega(\gamma,d),\gamma)$. The distance-dependence of community similarity is entirely embedded in the distance-dependence of the neighborhood occurrence density $\Omega(\gamma,d)$ for each species (distance does not appear anywhere else in the formula than in *Ω*, so that the functional form of the distance-dependence of similarity is entirely determined by the functional form of the decay of $\Omega(\gamma,d)$with distance. $\psi(an\Omega(\gamma,d),\gamma)$ at large distances is a decreasing function of aggregation, so that more aggregated communities are expected to display lower similarity values at large distances. $\psi(an\Omega(\gamma,d),\gamma)$at any distance increases with landscape-scale abundance *n* (or equivalently sample area *a*), so that high similarity values across the range of distances are expected when species have high landscape-scale abundances (or sampled with large sample areas), while low similarity values across the range of distances are expected when species have low landscape-scale abundances.

In a hypothetical community where all the species have same landscape-scale abundance *n* and aggregation γ, the slope of the distance-decay relationship at a given distance is expected to vary as $an\frac{\partial\Omega(\gamma,d)}{\partial d}\psi'(an\Omega(\gamma,d),\gamma)$ (Here *ψ’* is the derivate of *ψ*). *ψ’* is expected to be a decreasing function, making it difficult to predict how the slope varies with *an* (the slope is a product of an increasing function and a decreasing function of the product *an*). We would need to assume a functional form for *ψ* to assess the effect of sample area and landscape-scale abundances on the slope of the relationship. However, in the three tropical forests studied in the manuscript, results presented in the section *Empirical Evaluation* suggest that landscape-scale abundances and sample area have a weak influence on the rate of decay, except possibly for extreme values of landscape-scale abundances or sample areas.

**APPENDIX C. Poisson Cluster Process derivations**

Here, basing upon Cressie (1993), we derive a species occurrence probability $\psi(a,n,\gamma)$and *Ω*(*d*) under a Poisson Cluster Process with parameters *ρ*, *σ* and *μ* and bivariate Gaussian Kernel *h*.

**C1. Occurrence probability**

Let *N* be any point process on space *X*. The probability generating function of *N* is defined by:

$$G_{N}(\omega)\equiv E\left[\exp\left\{\int_{X} \log\omega(s)N(ds)\right\}\right]$$

Where $\omega$is any function $X\to[0,1]$

Let *t* be any real number, $x\in X$, and$\omega_{t}(x)=1-(1-t)\delta(x\in A)$ $G_{N}(\omega_{t})\equiv E\left[\exp\left\{\int_{A} \log tN(ds)\right\}\right]=E(t^{N(A)})$

Therefore, $t\to G_{N}(\omega_{t})$ is the generating function of $N(A)$

And $P(N(A)=0)=G_{N}(\omega_{t})|_{t=0}$

The occurrence probability in *A* is:

$\psi(a,n,\rho,\sigma)=1-P(N(A)=0)=1-G_{N}(\omega_{t})|_{t=0}$ [C.1]

The probability generation function of the Poisson Cluster Process defined in the manuscript is (directly derived from Cressie 1993):

$G_{N}(\omega)=\exp\left\{-\rho\int_{X} \left[1-\eta\left\{\int_{X} \omega(u+s)h(u)du\right\}\right]ds\right.$ [C.2]

Where $\eta$is the probability generating function of the number of events per cluster.

With $\omega_{t}(x)=1-(1-t)\delta(x\in A)$ $\int_{X} \omega_{t}(u+s)h(u)du=\int_{X} \omega_{t}(u)h(u-s)du=\int_{\bar{A}} h(u-s)du\int+t\int_{A} h(u-s)du$

Equation C.2 gives the generating function of $N(A)$:

$$G_{N}(\omega_{t})=\exp\left\{-\rho\int_{X} \left[1-\eta\left\{\int_{\overline{A}} h(u-s)du+t\int_{A} h(u-s)du\right\}\right]ds\right\}$$

For any *s* in *X*: $\int_{\overline{A}} h(u-s)du+\int_{A} h(u-s)du=\int_{X} h(u-s)du=1$

Thus, $G_{N}(\omega_{t})=\exp\left\{-\rho\int_{X} \left[1-\eta\left\{1+(t-1)\int_{A} h(u-s)du\right\}\right]ds\right\}$

The number of offspring per parent is Poisson-distributed with mean *μ*, so that: $\eta(t)=\exp(\mu(t-1))$

and $\eta\left\{1+(t-1)\int_{A} h(u-s)du\right\}=\exp(\mu(t-1)\int_{A} h(u-s)du)$

Thus,$G_{N}(\omega_{t})=\exp\left\{-\rho\int_{X} \left[1-\exp(\mu(t-1)\int_{A} h(u-s)du)\right]ds\right\}$

And from Equation C.1 we obtain the expression of the occurrence probability in a patch under a Poisson Cluster Process:

$$\psi(a,n,\rho,\sigma)=1-\exp\left\{-\rho\int_{X} \left[1-\exp(-\mu\int_{A} h(u-s)du)\right]ds\right\}$$

With $n=\rho\mu A_{0}$, we find that the occurrence probability in a sample of area A for a species’ with abundance *n* and parameters *ρ* and *σ* is given by:

$\psi(a,n,\rho,\sigma)=1-\exp\left[-anc(A)\right]$ [C.3]

With:

$c(A)=\frac{1}{\mu A}\int_{X} (1-\exp(-\mu\int_{A} h(u-s)du)ds$. [C.4]

The coefficient *c(A)* ranges between 0 and 1 and reflects clustering at sample scale *A*. It depends on *σ* and the number *μ* of individuals per cluster. The integral $\int_{A} h(u-s)du$ measures the probability that a “parent” with coordinate *s* gives rise to an “offspring” in sample *A*.

**C2. Omega statistic**

The derivation of Ripley’s K statistic under the Poisson Cluster Process is known (Cressie 1993; Plotk*in et a*l. 2000):

$K(d)=\pi d^{2}+\frac{1}{\rho}(1-\exp(-d^{2}/4\sigma^{2}))$ [C.5]

Combining Equation B.8 and Equation C.5 gives:

$$\Omega(d_{1,2})=1-\frac{1}{\pi\rho}[\frac{\exp(-\frac{d_{2}^{2}}{4\sigma^{2}})-\exp(-\frac{d_{1}^{2}}{4\sigma^{2}})}{d_{2}^{2}-d_{1}^{2}}]$$

Writing $D=d^{2}$, we find:

$$\Omega(D_{1,2})=1-\frac{1}{\pi\rho}[\frac{\exp(-\frac{D_{2}}{4\sigma^{2}})-\exp(-\frac{D_{1}}{4\sigma^{2}})}{D_{2}-D_{1}}]$$

$$\Omega(D)=\lim_{D_{1}\to D_{2}=D}\Omega(D_{1,2})$$

$\lim_{D_{1}\to D_{2}=D}\frac{\exp(-\frac{D_{2}}{4\sigma^{2}})-\exp(-\frac{D_{1}}{4\sigma^{2}})}{D_{2}-D_{1}}=f'(D)$with $f(x)=\exp(-\frac{x}{4\sigma^{2}})$

$$f'(x)=-\frac{1}{4\sigma^{2}}\exp(-\frac{x}{4\sigma^{2}})$$

So that $\Omega(D)=1+\frac{1}{4\pi\rho\sigma^{2}}\exp(-\frac{D}{4\sigma^{2}})$

And finally:

$\Omega(d)=1+\frac{1}{4\pi\rho\sigma^{2}}\exp(-\frac{d^{2}}{4\sigma^{2}})$ [C.6]

Combining Equation 2 in the manuscript with Equation C.3 and Equation C.6 lead to the derivation for the distance-decay relationship under the Poisson Cluster Process (Equations 4 to 6 in the manuscript):

$\chi(a,d)=\frac{\int(1-\exp(-anc(A)))(1-\exp(-anc(A)\Omega(\rho,\sigma,d)))\xi(n,\rho,\sigma)dnd\rho d\sigma}{\int(1-\exp(-anc(A)))\xi(n,\rho,\sigma)dnd\rho d\sigma}$ [C.7]

with $c(A)=\frac{1}{\mu A}\int_{X} (1-\exp(-\mu\int_{A} h(u-s)du)ds$

and $\Omega(\rho,\sigma,d)=1+\frac{1}{4\pi\rho\sigma^{2}}\exp(-\frac{d^{2}}{4\sigma^{2}})$

**C3. Relationship between Poisson Cluster Process coefficient of aggregation *c* and negative binomial clustering parameter *k***

The probability that a species with landscape-scale abundance *n* occurs in a sample which area constitutes a fraction *a* of the landscape is given by:

For the negative binomial distribution with parameter *k*:

$$\psi(a,n)=1-(1+\frac{an}{k})^{-k}$$

For the Poisson cluster process:

$$\psi(a,n)=1-\exp(-anc)$$

It follows: $(1+\frac{an}{k})^{-k}=\exp(-anc)$

$$-k\ln(1+\frac{an}{k})=-anc$$

And finally:

$c=\frac{k}{an}\ln(1+\frac{an}{k})$ [C.8]

Equation C.8 provides the exact analytical link between *c* and *k*.

**APPENDIX D. Figures relevant to empirical evaluation of *Qualitative predictions* in BCI, Yasuni and Korup**


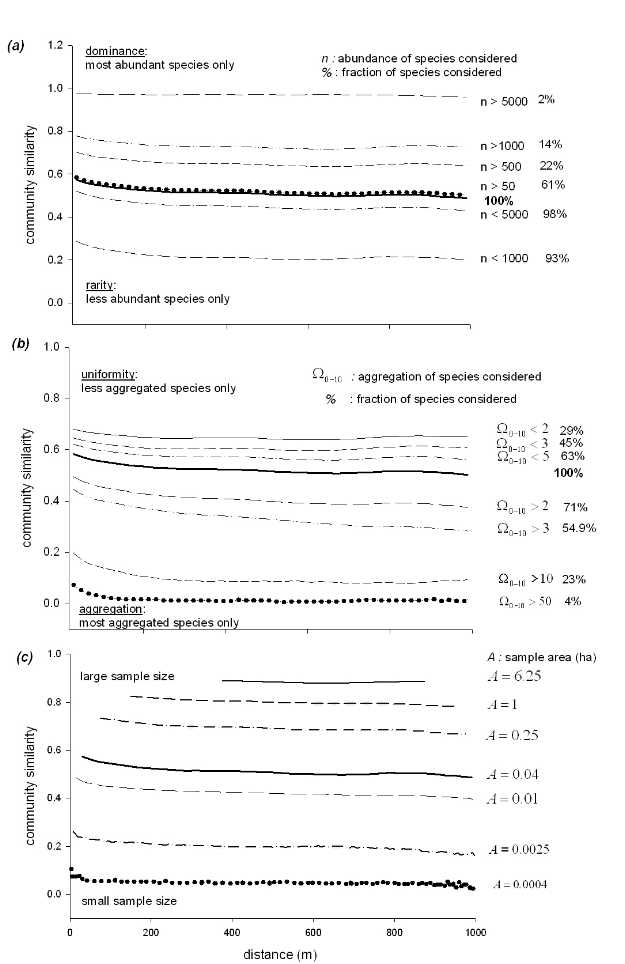
**Figure D1. Empirical evaluation in BCI**

**Figure D2. Empirical evaluation in Yasuni**

**
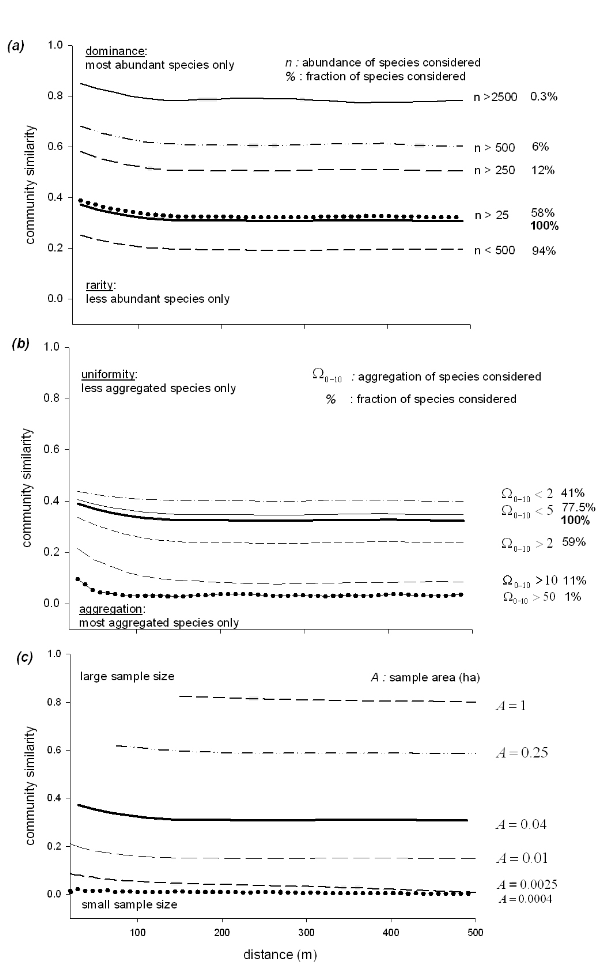
**

**Figure D3. Linear-log plots emphasizing the importance of aggregation in shaping the distance-decay relationship in Korup**

**
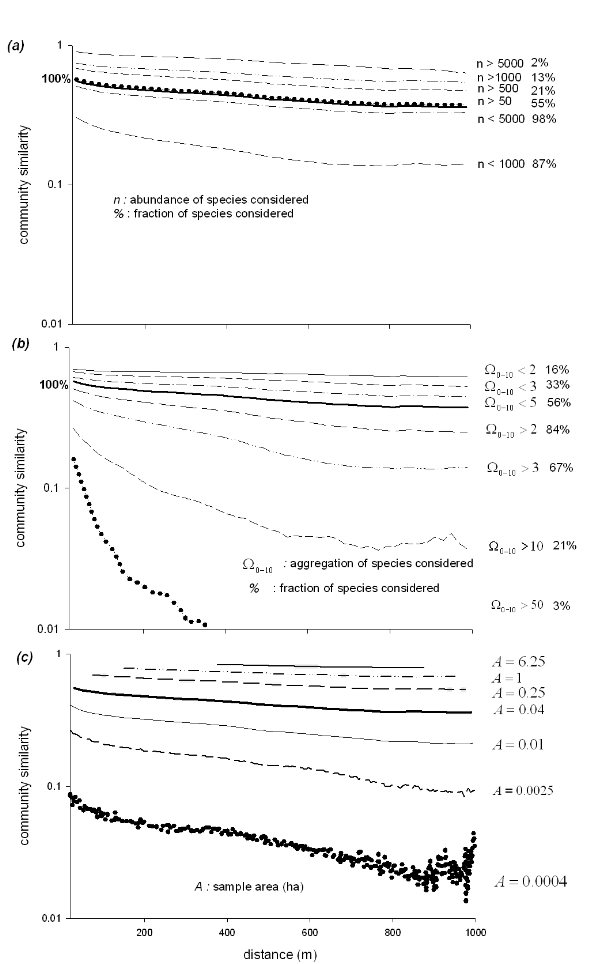
**

**APPENDIX E. Estimation and distribution of Poisson Cluster Process parameters**

All methods presented here are derived from Cressie (1993), Plotkin (2000) and Diggle (2003).

**E1. Estimation of Poisson Cluster Process parameters**

For each species in a forest, we first estimated the parameters *ρ* and *σ* as described below, and then deduced the parameter *μ* from the relationship$n=\rho\mu A_{0}$. For reasons explained below, we treated differently species with less or more than 20 individuals in the plot. Species with more than twenty individuals represented 63% of the pool of species in Yasuni, 72% in BCI, and 66% in Korup.

*a*) For species with more than 20 individuals in the plot, the parameters *ρ* and *σ* were estimated by best-fit of the observed Ripley’s *K* curve $K(d_{1}),...,K(d_{\max})$ to the expected$K^{PCP}(d_{1}),...,K^{PCP}(d_{\max})$, where$K^{PCP}(d)=\pi d^{2}+\frac{1}{\rho}(1-\exp(-d^{2}/4\sigma^{2}))$.

The best fit was found by minimizing the integral $\int_{0}^{d_{\max}} (K(h)^{c}-(K(h)^{PCP})^{c})^{2}dh$ with $c=0.25$, based on Plotkin *et al.* (2000). Following Plotkin *et al.* (2000), *d_max_* was chosen based on the observation of the curves describing *P_d_-P_rand_,* where *P_d_* is the proportion of trees in the plot, distance *d* apart, which are the same species, and *P_rand_* is the expected value of *P_d_* under random placement. These curves suggested choosing 200 ≤ *d_max_* ≤ 400. We performed all analyses (fitting the parameters of the Poisson Cluster Process and calculating the corresponding distance-decay relationships), for *d_max_*= 200, *d_max_* = 300, and d_max_ = 400. Choosing a different value for *d_max_* did not change the resulting distance-decay curves significantly, and we reported in the manuscript results obtained with the intermediate value *d_max_* = 300. However, we do not exclude the possibility that the fit of the Poisson Cluster parameters with *d_max_* chosen specifically species by species would improve the community level fit of the distance-decay.

*b*) When the spatial distribution of species is close to random, the value of the estimated parameters can be unrealistic. When we found a value for mean cluster diameter larger than the length of the plot, we set this value to the length of the plot. When we found more clusters than individuals in the plot, we set the number of clusters to the number of individuals in the plot. These arbitrary values were designed to reproduce the random distribution of the species and are not expected to influence our results. Reasonable parameter estimations were found for 74% of the species with more than 20 individuals in Yasuni, 91% in BCI and 95% in Korup. In Figures 4 and 5 in the manuscript, we consider theses species only.

*c*) We consistently observed across forests that, for a high proportion of species with less than 20 individuals, fitting Ripley’s *K* curve did not lead to a realistic estimation of the parameters. We therefore arbitrarily chose *n* = 20 as a cut-off above which we did not use the statistical estimation technique described above. Instead, we estimated the parameters for rare species using those estimated in *a*) for species with more than 20 individuals and realistic parameter values. We assigned *ρ* and *σ* values to species with less than 20 individuals based on the log-log regression between *ρ* and *n* and the log-log regression between *σ* and *n* (Figure 5 in the manuscript). Log-log regressions were suggested by the log-normal shape of the parameters distributions (Figures 4 and 6). This estimation for rare species is rough and concerns a high fraction of the species in the plot. However, the rare species are not expected to influence distance-decay relationships strongly (Figure 3 in the paper and Appendix D). We tested the possibility that the bad fit of the Poisson Cluster Process distance-decay relationship in Korup to the observed curve results from a bad estimation of the Poisson Cluster Process parameters for rare species. We performed in Korup distance-decay analyses similar to those presented in Figure 6 but excluding the rare species and found, consistently with Figure 6, that the curves obtained with the Poisson Cluster Process did not fit the observed curves. .

**E2. Distributions of mean clump radius**
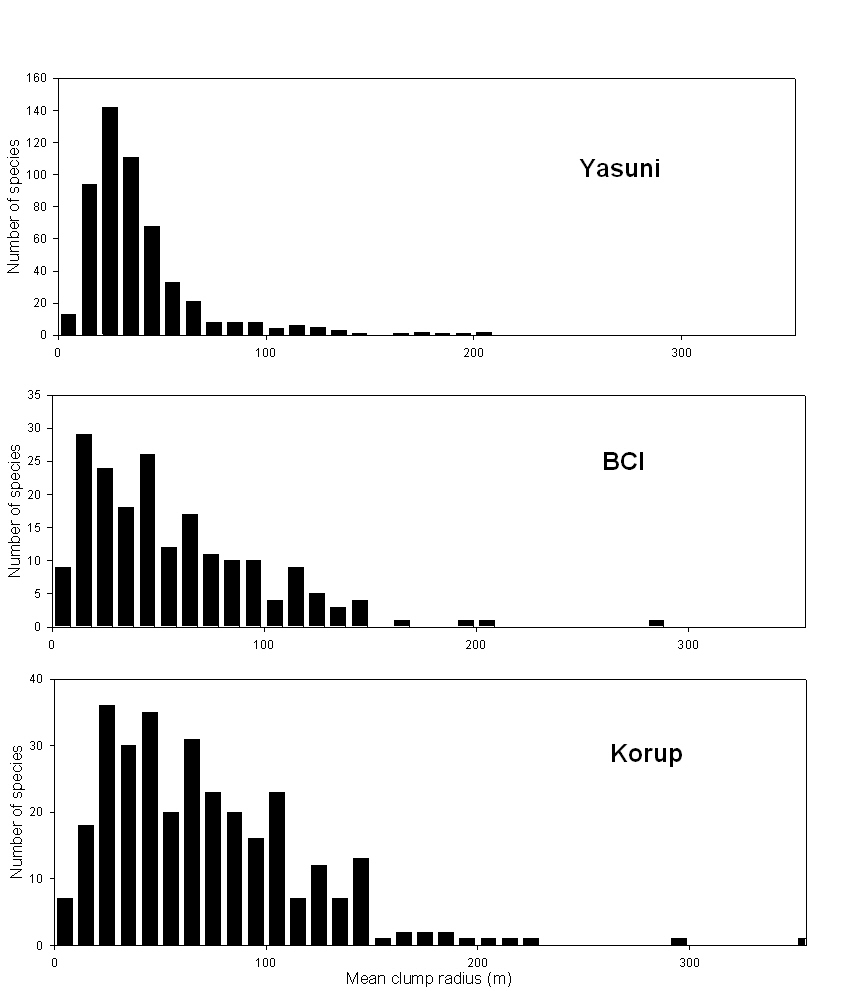
**on a linear-linear scale**

**APPENDIX F. Supplementary information relevant to “Empirical evaluation”**

**F1. Comparing measures of aggregation**

Intuitive interpretation of *c*

*c(A)* is a coefficient ranging between 0 and 1 that controls the deviation of the occurrence probability from the one expected under random placement ($\psi\left(an\right)=1-e^{-an}$). Values of *c*(*A*) close to 0 correspond to high clustering. Values of *c*(*A*) close to 1 correspond to random placement. Intuitively, the number of individuals in a population should be multiplied by $\frac{1}{c(A)}$ to obtain an occurrence probability corresponding to random placement. *c(A)* is scale dependent, and depends on *μ* and *σ* only:

$$c(A)=\frac{1}{\mu A}\int_{X} (1-\exp(-\mu\int_{A} h(u-s)du)ds$$

For *σ* constant, aggregation increases (according to *c*) if and only if the density of individuals per cluster *μ* increases.

Intuitive interpretation of *Ω*

*Ω* is a decreasing function of distance that reflects the deviation of the neighborhood occurrence probability from the occurrence probability. High values of *Ω* at small distances and steep decays of *Ω* correspond to high clustering. Under random placement, *Ω* is equal to 1 at any distance. Intuitively, *Ω* measures the effect of being close to a conspecific. It depends on *ρ* and *σ* only:

$$\Omega(\rho,\sigma,d)=1+\frac{1}{4\pi\rho\sigma^{2}}\exp(-\frac{d^{2}}{4\sigma^{2}})$$

At constant *σ*, aggregation decreases (according to *Ω*) if and only if the density of clusters increases. Note that, despite the fact that *Ω* is designed to investigate local densities around conspecifics, it is independent of the number of individuals per cluster (*μ*). Note also that *Ω* values in adjacent distance classes are highly correlated (Cond*it et a*l. 2000), justifying measuring Ω values in the arbitrary 0-10 m class. This choice is not expected to influence our results.

When abundance increases, both the number of clusters and the number of individuals per cluster increase (Figure 5 in the paper). The increase in number of clusters tends to reduce aggregation, while the increase in number of individuals per cluster tends to increase aggregation. *σ*, *Ω* and *c* emphasize different aspects of aggregation (size, number or density of clusters), each of which scale differently with abundance.

**F2. Testing Equation 4 against simulations and the Poisson Cluster Process against data**

Simulating the Poisson Cluster Process in each forest

Once the parameters *ρ* and *σ* are estimated for each species in a forest, the Poisson Cluster equivalent of the forest is obtained by overlaying each independently simulated species. To avoid edge effects, each species is simulated according to the Poisson Cluster in a larger area surrounding the actual plot, corresponding here to a 750 hectare plot surrounding the 50 hectare plot in Korup and BCI and the 25 hectare plot in Yasuni. We tested that increasing the area surrounding the plots did not influence the results.

Testing the Poisson Cluster Process against data

*Discrete equivalent of Equation 4*

To compute expected Poisson Cluster Process similarity values (Eq. 4) against data, we worked with the discrete equivalent of Equation 4, which reads:

$\chi(a,d)=\frac{\sum_{i=1}^{S_{0}} (1-\exp(-an_{i}c_{i}(A)))(1-\exp(-an_{i}c(A)\Omega(\rho_{i},\sigma_{i},d)))}{\sum_{i=1}^{S_{0}} (1-\exp(-an_{i}c_{i}(A)))}$ [F.1]

with

$$c_{i}(A)=\frac{1}{\mu_{i}A}\int_{X} (1-\exp(-\mu_{i}\int_{A} h_{i}(u-s)du)ds$$

$h_{i}(x,y)=\frac{1}{2\pi\sigma_{i}^{2}}\exp(-\frac{(x^{2}+y^{2})}{2\sigma_{i}^{2}})$

The discrete equivalent for the species-area relationship is given by the denominator of Equation F2.1

*Expectation of Sørensen similarity under random placement*

Expectations under random placement were computed using Plotkin & Muller-Landau’s (2002) sampling formula: under random placement, $\psi*=\psi$ at any distance (or equivalently $\Omega(d)=1$at any distance), and $\chi$is equal to the average similarity across the landscape at any distance. The discrete equivalent of equation B.1 reads, for $a=b$:

$\chi(a)=\frac{\sum_{i=1}^{S_{0}} \psi(an_{i},\gamma_{i})^{2}}{\sum_{i=1}^{S_{0}} \psi(an_{i},\gamma_{i})}$

Under random placement: $\psi(an_{i},\gamma_{i})=1-\exp(-an_{i})$

and:

$\chi(a)=\frac{\sum_{i=1}^{S_{0}} (1-\exp(-an_{i}))^{2}}{\sum_{i=1}^{S_{0}} (1-\exp(-an_{i}))}$ [F.2]

*Species-area and distance-decay relationship 95% confidence intervals*

The 95% confidence intervals of the species-area and distance-decay relationships are obtained by Monte-Carlo simulations of Poisson Cluster Process communities. The 95% confidence envelop is composed of the 38 out of 40 intermediate values obtained by simulations at each area (species area relationship) or distance class (distance-decay relationship).

*Test of Poisson Cluster Process hypotheses and sensitivity analyses*

We test, for each forest, the null hypothesis H0 that population aggregation can be characterized by the Poisson Cluster Process, using both species-area and distance-decay curves. Below, we describe this test for the distance-decay relationship. The approach for species-area curves is similar, replacing community similarity by species richness, and distances by areas.

We use a test inspired from spatial statistics (Diggle 2003; Gre*en et a*l. 2003): we measure the deviation of the observed similarity values from the theoretical expectations under the Poisson Cluster Process, using:

$$k_{observed}=\sum_{i} [\chi_{observed}(d_{i})-\chi_{predicted}(d_{i})]^{2}$$

where$\chi(d_{i})$is the average similarity in distance-class *i.*

We also measure, for 40 simulated communities, the deviation of the similarity values from the theoretical expectations under the Poisson Cluster Process, using:

$k_{simulated}=\sum_{i} [\chi_{simulated}(d_{i})-\chi_{predicted}(d_{i})]^{2}$.

The null hypotheses H0 that a distance-decay curve arise form the Poisson Cluster Process is rejected at the 5% level confidence if $k_{observed}>k_{simulated}$ for at least 38 out of the 40 simulations.

With these criteria, H0 is not rejected with the species-area relationship in any of the forests. It is rejected in all three forests with the distance-decay relationship, except in Yasuni and BCI for the 25  × 25 meter sample size.

**Figure F1. Comparison of theory with data in Yasuni, BCI, and Korup for different sample sizes**

Thick solid lines represent observed curves. Thin solid lines represent curves predicted by the Poisson Cluster Process (Eq. F.1 above). Thin dotted lines represent *95%* confidence intervals produced by simulation of the Poisson Cluster Process. Dashed lines represent curves predicted from the species abundance distribution in each forest assuming random placement (Eq. F.2 above). The Poisson Cluster Process improves predictions under random placement in the three forests, and offers a good first approximation in Yasuni and BCI. The process tends to overestimate similarity values in the forests for small sample sizes, and to underestimate them for larger sample sizes. In Korup, the Poisson Cluster Process fails to reproduce the shape of observed distance-decay curves, and largely overestimate similarity values, particularly for the smallest sample sizes.


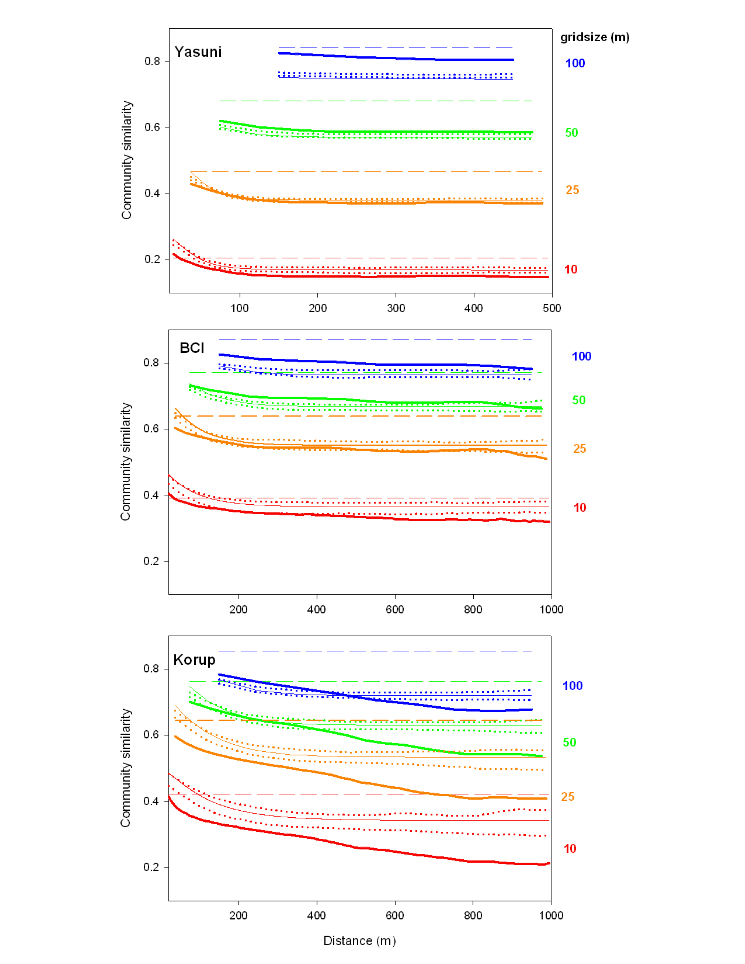
**Figure F1.**

**APPENDIX G. Supplementary Material References**

*Condit R., Ashton P.S., Baker P., Bunyavejchewin S., Gunatilleke S., Gunatilleke N., Hubbell S.P., Foster R.B., Itoh A., LaFrankie J.V., Seng L.H., Losos E., Manokaran N., Sukumar R. & Yamakura T. (2000) Spatial patterns in the distribution of tropical tree species. Science, 288, 1414-1418*

*Cressie N.A.C. (1993) Statistics for Spatial Data. Winey & Sons, New-York.*

*Diggle P.J. (2003) Statistical Analyses of Spatial Point Patterns. 2nd edn. Edward Arnold, London.*

*Green J.L., Harte J. & Ostling A. (2003) Species richness, endemism, and abundance patterns: tests of two fractal models in a serpentine grassland. Ecology Letters, 6, 919-928*

*Plotkin J.B. & Muller-Landau H.C. (2002) Sampling the species composition of a landscape. Ecology, 83, 3344-3356*

*Plotkin J.B., Potts M.D., Leslie N., Manokaran N., LaFrankie J. & Ashton P.S. (2000) Species-area curves, spatial aggregation, and habitat specialization in tropical forests. J. Theor. Biol., 207, 81-99*
